# Psychometric properties and factor structure of the satisfaction with life scale in Ecuadorian university students

**DOI:** 10.3389/fpsyg.2025.1536973

**Published:** 2025-03-20

**Authors:** Victor Manuel López-Guerra, Tatiana Isabel Pucha-Loarte, Luisa T. Angelucci, Pablo V. Torres-Carrión

**Affiliations:** ^1^Department of Psychology, Universidad Técnica Particular de Loja, Loja, Ecuador; ^2^Faculty of Humanities and Education, Universidad Católica Andrés Bello, Caracas, Venezuela; ^3^Department of Computer Science, Universidad Técnica Particular de Loja, Loja, Ecuador

**Keywords:** psychometric properties, factor structure, satisfaction with life, college students, Ecuador

## Abstract

**Background:**

The Satisfaction with Life Scale (SWLS) measures the global cognitive judgment about one’s own life. Although it has been validated in different countries and populations, in the Ecuadorian context, it has not been validated for a university population.

**Objective:**

The present study aimed to analyze the psychometric properties of the SWLS in a large sample of Ecuadorian college students.

**Methods:**

Instrumental-psychometric study with a sample of 4,524 participants from three Ecuadorian universities, with an average age of 22 (±3.15). The SWLS was evaluated in terms of its factor structure, factorial invariance, internal consistency and correlations with other measures related to mental health.

**Results:**

The SWLS factor structure optimally fits the single-factor model (*X^2^/df* = 3.79; CFI = 0.998; TLI = 0.996; SRMR = 0.023; RMSEA = 0.030). This model is invariant between men and women. The internal consistency of this instrument is acceptable according to the omega coefficient (*ω* = 0.84). The scores of this scale correlate, as expected, negatively with constructs such as depression and stress.

**Conclusion:**

The SWLS is a valid and reliable instrument to assess the personal perception of life satisfaction of Ecuadorian university students.

## 1 Introduction

Positive psychology scientifically studies positive human functioning, incorporating all those concepts concerning happiness, well-being and optimistic approaches to mental health (Compton and Hoffman, 2019). Among this range of components, human well-being has been the subject of special study, approached from two research perspectives that come from ancient philosophical movements (Cooke et al., 2016): the eudaimonic that is linked to the development of human potential and refers to psychological well-being (Garassini, 2021; Ryan and Deci, 2001) and the hedonic, which is fundamentally linked to happiness and refers to subjective well-being (Oxholm and Paldam, 2019).

In recent years, there has been an increase in research on subjective well-being, due to its direct implication with the health and longevity of individuals (Areepattamannil and Bano, 2020). Subjective well-being contains two elements: the affective component, which refers to the pleasant and unpleasant feelings experienced in everyday situations, and the cognitive component, called satisfaction with life (Jiménez et al., 2019).

Satisfaction with life is the conscious global assessment or judgment that a person makes of all the domains of their life (family, friends, studies, work, health, income), comparing the actual circumstances they have experienced with self-imposed standards perceived as appropriate or inappropriate (Diener et al., 1985; Diener et al., 2017; Kjell and Diener, 2021). The evaluative nature of satisfaction with life allows for a broad view of the degree of pleasure/enjoyment that the individual has with their life, and, depending on how positive or negative it is, it becomes a protective or risk factor for human well-being (Véliz Burgos et al., 2017).

Satisfaction with life is a construct that is associated with numerous variables; it is positively related to other indicators of emotional well-being such as high self-esteem, traits of joy, emotional/behavioral regulation strategies and resilience (Chilicka et al., 2020), and negatively related to psychosocial factors that deteriorate mental health such as perception of loneliness, violent behavior, drug use (Garrido-Montesinos et al., 2018), anxiety, depression (Seo et al., 2018), suicidal ideation (Dirzyte et al., 2021) and stress (Meule and Voderholzer, 2020; Nooripour et al., 2023).

Specifically, satisfaction with life has been studied in the Ecuadorian population. Avila and Cañas-Lucendo (2023) found a low level of satisfaction in adolescents (more than 60% dissatisfied and very dissatisfied). However, in university students, Hidalgo-Fuentes et al. (2021) and Moreta-Herrera et al. (2018) found medium-high levels of satisfaction, although the latter authors highlight a significant percentage of risk in satisfaction with life (low scores: 19%). Regarding differences in satisfaction by sex, there are inconsistencies, only Moreta-Herrera et al. (2018) found that women presented more satisfaction than men. In adults, Arias and García (2018) indicate high levels of satisfaction, where men presented greater satisfaction with life than women. Older adults have medium levels of satisfaction with life, being higher in those who live in urban areas and have a higher level of education (Ubilla et al., 2020).

Given the evidence of the relevant role that satisfaction with life plays in mental health, it is necessary to create tools that allow its effective assessment. Initially, instruments such as the Life Satisfaction Index by Neugarten et al. (1961) and the Philadelphia Moral Scale by Lawton (1975) were designed, but they were ineffective because the difficulties in their design prevented the unidimensional evaluation of the construct and limited their application to different populations such as, for example, the geriatric population. Based on these limitations, the Satisfaction with Life Scale (SWLS) by Diener et al. (1985) was developed, which offers a comprehensive, stable and effective assessment perspective by having items that are strongly consistent in most cultures (Diener et al., 2017), having made it the most widely used tool worldwide (Vinaccia Alpi et al., 2019).

In the development phase of the Satisfaction with Life Scale, Diener et al. (1985) designed a battery of 48 items that included the main dimensions of subjective well-being; they performed an exploratory factor analysis that allowed the items to be grouped into three specific factors: satisfaction with life, positive affects, and negative affects. Considering that the objective was to specifically evaluate satisfaction with life, they selected only the items that were grouped in this factor whose loadings were > 0.60 and subjected them to a semantic similarity analysis. As a result, they obtained a 5-item scale with seven Likert-type response options ranging from 1 “completely disagree” to 7 “completely agree” (Diener et al., 1985).

To validate the Satisfaction with Life Scale, Diener et al. (1985) administered the instrument to a sample of 339 students from the University of Illinois in the United States and randomly re-administered it 2 months later to 76 participants. To verify the construct validity, they carried out an exploratory and confirmatory factor analysis that allowed them to identify a single-factor structure that represents 66% of the total variance of the construct. Reliability was analyzed using the alpha coefficient and the test–retest coefficient, whose values were *α* =0.87 and *r* = 0.82, respectively, indicating high reliability. Finally, to determine concurrent validity, they applied other scales that also measured subjective well-being together with the SWLS and, through Pearson’s correlation coefficient, they found moderate and significant correlations with the scores of: Fordyce’s Global Scale (*r* = 0.58; *p* < 0.001), Fordyce Happiness Measure (*r* = 0.58, *p* < 0.001), Cantril Self-Assessment Scale (*r* = 0.62, *p* < 0.001), Gurin Scale (*r* = 0.9, *p* < 0.001), Canverse and Rodger’s General Well-Being and Affect Inventory (*r* = 0.75, *p* < 0.001) and with Bradburn’s Positive Affect Measure (PAS; *r* = 0.50, *p* < 0.001; Diener et al., 1985). Based on the results obtained, they determined that the SWLS had optimal psychometric properties.

Subsequently, Atienza et al. (2000) analyzed the psychometric properties of the original scale translated into Spanish in Valencia, Spain, in a sample of 697 adolescents aged 11, 13, and 15 years. The authors found that the scale had satisfactory internal consistency (*α* = 0.84) and a one-factor structure that explained 53.7% of the total variance of the construct. In addition, this study revealed that, in terms of convergent and divergent validity, the scale maintains positive and statistically significant associations with academic satisfaction (*r* = 0.32, *p* < 0.001) and feelings of happiness (*r* = 0.46, *p* < 0.001); and a negative and statistically significant association with feelings of loneliness (*r* = −0.31, *p* < 0.001).

In the last 5 years, a series of studies have been developed that have validated the scale in different areas and populations, in various parts of the world such as Lithuania (Dirzyte et al., 2021), Sweden (Garcia et al., 2021), Spain (Merino et al., 2021), India (Areepattamannil and Bano, 2020) and Iran (Nooripour et al., 2023), and in Latin American countries such as Chile (Bagherzadeh et al., 2018), Puerto Rico (González Rivera and Rosario Rodríguez, 2020), Argentina (Mikulic et al., 2019), Colombia (Álvarez-Merlano and Castro-Bocanegra, 2022; Vinaccia Alpi et al., 2019), Ecuador (Arias and García, 2018; Schnettler et al., 2017), Peru (la Cruz et al., 2018) and the Dominican Republic (Zerpa et al., 2023).

Some of these studies report a single-factor structure, but when examining the items, the presence of correlated errors has been found between items one and two (Bagherzadeh et al., 2018; la Cruz et al., 2018; Sachs, 2003) and items four and five (la Cruz et al., 2018; Jovanović, 2016; Moksnes et al., 2014). Also, a two-factor factor structure has been found, where items 1, 2, and 3 load on the first factor and assess present achievements of satisfaction, while items 4 and 5 load on the second factor and focus on past achievements (Bagherzadeh et al., 2018; Pavot and Diener, 1993; Sachs, 2003). These differences in the factor structure could be due to cultural differences. Regarding factorial invariance, there is no conclusive evidence regarding gender differences in the Spanish version of SWLS (Bagherzadeh et al., 2018; la Cruz et al., 2018).

In Ecuador, the psychometric properties of the SWLS have been evaluated in adults and seniors (Arias and García, 2018; Schnettler et al., 2017), but there are no studies with young people. Since young people experience stressful situations that can decrease their levels of satisfaction with life (Gil Roales-Nieto and Segura Sánchez, 2016), it is essential to validate this scale in such a population.

Therefore, the objective of this research is to analyze the psychometric properties, factor structure and factorial invariance of the Satisfaction with Life Scale (SWLS) in Ecuadorian university students. The following hypotheses are proposed:

The scale presents a single-factor structure in the population of Ecuadorian university students.

There is factorial invariance according to the sex of the participants.

The SWLS shows adequate levels of reliability and validity in this sample.

## 2 Materials and methods

### 2.1 Type of study and research design

A study was conducted with an instrumental-psychometric design aimed at validating a psychological assessment instrument (Montero and León, 2002).

### 2.2 Participants

The population was comprised of in-person university students from three universities in Ecuador: Technical University of the North, Private Technical University of Loja, and Salesian Polytechnic University. The sample consisted of 4,524 students, selected by non-probabilistic convenience sampling; the mean age of the participants was 22 (SD = 3.15), 55% were women, 94% were single, 90% were full-time students, and in terms of ethnicity, 90% were mestizos, 5% were indigenous, 2% were Afro-Ecuadorians, and 1.2% were white.

### 2.3 Measures

*Sociodemographic data*. This section included information regarding basic sociodemographic data, such as age, sex, marital status and geographic region.

*Satisfaction with Life Scale (SWLS)*. This instrument was designed by Diener et al. (1985) and adapted to Spanish by Atienza et al. (2000). It measures or assesses global cognitive judgments about satisfaction with life. It consists of 5 items that include a 7-point Likert-type response scale, ranging from 1 = “completely disagree” to 7 = “completely agree.” The total score ranges from 5 to 35, with higher values indicating greater satisfaction with life. The score is categorized according to the following reference points: 5–9 “extremely dissatisfied”; 10–14 “dissatisfied”; 15–19 “slightly dissatisfied”; 20 “neutral”; 21–25 “slightly satisfied”; 26–30 “satisfied”; 31–35 “extremely satisfied” (Diener et al., 1985). The study of the analysis of the psychometric properties of the scale adapted to Spanish shows that the instrument has good internal consistency (*α* = 0.84), a single-factor structure that explains 66% of the total variance of the construct and satisfactory convergent and divergent validity with other psychological variables such as academic satisfaction, feelings of happiness and feelings of loneliness (Atienza et al., 2000).

*Patient Health Questionnaire (PHQ-9)*. The instrument was designed by Kroenke et al. (2001) to assess depressive symptoms according to DSM-IV criteria during the previous 2 weeks. The scale has 9 items that include a 4-point Likert-type scale for response: 0 “not at all”; 1 “several days”; 2 “more than half the days”; 3 “almost every day.” The total score can vary from 0 to 27 points that can be included in 5 categories of severity of the depressive disorder: 0–4 “none”; 5–9 “mild”; 10–14 “moderate”; 15–19 “moderately severe” and 20–27 “severe.” Regarding its psychometric properties, according to a study carried out on Ecuadorian university students, the scale presents adequate internal consistency (*ω* = 0.90), a hierarchical structure that includes a general factor and three latent factors: somatic, cognitive/affective and concentration/motor; the scale also possesses satisfactory convergent and divergent validity with several health indicators (López-Guerra et al., 2022). Medium to strong negative correlations were expected between PHQ-9 and SWLS scores.

*Perceived Stress Scale (PSS-10)*. The original scale was developed by Cohen et al. (1983). The 10-item version, adapted to Spanish by Remor (2006), assesses the degree to which people perceive a lack of control in their daily lives over the previous month. The 10 items on the scale provide five response options: 0 “never,” 1 “almost never,” 2 “occasionally,” 3 “often” and 4 “very often.” To tally the result, the scores for the following items are inverted: 4, 5, 7 and 8, and finally the scores for the 10 items are added together. Higher scores indicate higher levels of perceived stress and vice versa for lower scores. Regarding the psychometric properties of the scale, in a study where it was linguistically and culturally adapted to Ecuador, good internal consistency was found (*α* = 0.85 ω = 0.87), a bifactorial structure that explains 56.99% of the total variance, as well as satisfactory convergent validity with multiple health indicators (Ruisoto et al., 2020).

### 2.4 Procedure

This study was conducted within the framework of a larger project to predict drug use in university students from three institutions in Ecuador. The study was approved by the Human Research Ethics Committee (Comité de Ética de Investigación en Seres Humanos, - CEISH March 6, 2019) of the Private Technical University of Loja - UTPL, Ecuador (UTPL-DIS-2019-0088-O) and was conducted according to the principles expressed in the Declaration of Helsinki (Association, W M, 2013). Digital informed consent was obtained from all participants, who received feedback on the results of their assessment.

For the development of the study, students from three universities in Ecuador were initially invited by email to participate in the research. For 10 weeks, a process of awareness and communication was carried out aimed at students using internal institutional media, social media of the universities and campaigns on the university campuses of the three institutions in order to publicize the research and the collection of data from students who wished to participate simultaneously proceeded. The application of the instruments was carried out online, average response rate across universities was 47.80%, ranging from 39.10 to 56.10%.

### 2.5 Data analyses

Statistical analyses were performed using IBM Statistical Package for the Social Sciences (SPSS) software, version 24 (IBM Inc., Chicago, IL, United States) and the JASP program, version 0.18.3.0.

First, the factor structure was analyzed by performing an exploratory factor analysis (EFA) and a confirmatory factor analysis (CFA). Following Harrington (2009) recommendation that indicates that, to obtain the factor structure, both the EFA and the CFA should be performed in different samples, the total sample (*N* = 4,524) was divided into two independent and homogeneous random samples, subsamples nA = 2,264 and nB = 2,260. The chi-square statistical test did not reveal significant differences in both subsamples, so the random selection helped to maintain the same proportion of sociodemographic characteristics in each of them.

The first subsample (nA) was used to perform an EFA in order to determine the adequacy of the factor loading in each item of the SWLS and the factor structure of the scale was analyzed. For this, the feasibility of carrying out an EFA was previously evaluated using the Kaiser-Meyer-Olkin (KMO) factor adequacy criterion and Bartlett’s sphericity test, where values ≥ 0.80 for the former (Kaiser, 1970) and significance levels *p* < 0.05 for the latter (López-Aguado and Gutiérrez-Provecho, 2019) indicate the interrelation of the data. For the EFA, the principal axis factorization method was used in combination with oblimin rotation, retaining factor loadings greater than 0.40 in the rotated matrix (Lloret-Segura et al., 2014).

The second subsample (nB) was used to perform a CFA. Considering that SWLS is in Likert format and is an ordinal measure, a diagonal weighted least squares (DWLS) estimation method using polychoric correlations was used to perform the CFA. The DWLS method is recommended for large samples (N > 200; Freiberg Hoffmann et al., 2013). The indices selected to assess the goodness of fit of the studied models were the chi-square (χ^2^) ratio by degrees of freedom (*df*), Bentler comparative fit index (CFI), Tucker-Lewis index (TLI), standardized root mean square residual (SRMR), and the root mean square error of approximation (RMSEA). To examine the adequacy of the model, the following parameters were considered: X^2^/df ≤ 3 adequate, ≥ 2 optimal (Byrne, 2016); CFI and TLI ≥ 0.90 adequate, ≥ 0.95 optimal; RMSEA and SRMR ≤0.08 adequate, ≤ 0.05 optimal (Hu and Bentler, 1999).

Second, the average variance extracted (AVE) was calculated in order to assess whether a set of indicators really measures a given construct and are not measuring another different concept. The acceptance criterion is that the average variance extracted (AVE) of a construct must be greater than 0.5, meaning that the construct shares more than half of its variance with its indicators, with the rest of the variance being due to measurement error (Fornell and Larcker, 1981).

Third, factorial invariance was assessed for the total, male, and female samples in the second subsample (nB), taking into account the following models: configural invariance (M1), indicating an unrestricted factor structure (baseline); metric invariance (M2), where equivalence restrictions between factor loadings are established; scalar invariance (M3), that is, loading and intercept equivalence restrictions; and strict invariance (M4), considering the equivalence restrictions of factor loadings, intercepts, and residuals. Measurement invariance and its levels were evaluated according to the recommendations of Cheung and Rensvold (2002): ΔCFI ≤0.01 and ΔRMSEA ≤0.015.

Fourth, the reliability of the scale was analyzed through internal consistency analysis, using the McDonalds omega coefficient (*ω*), considering values ≥ 0.70 as satisfactory (Nunnally, 1978). The use of this coefficient was considered since it can be used in ordinal, unidimensional scales, it does not depend on the number of items and, when working with the factor loadings, it makes the calculations more stable. In addition, it is an adequate measure of reliability if the tau equivalence principle is not met, which is often violated in practice (Ventura-León and Caycho-Rodríguez, 2017; Vizioli, 2021).

Fifth, divergent validity was analyzed based on the Pearson correlation (r) between SWLS scores and scores on the depression (PHQ-4) and perceived stress (PSS-10) scales, expecting negative, moderate, and strong correlations with a significance level of *p* < 0.05. Cohen’s (1988) recommendations were used to establish the magnitude of the relationship between variables, thus *r* = 0.10 represents a weak or small association, *r* = 0.30 is considered a moderate relationship, and *r* ≥ 0.50 represents a strong or large correlation.

Finally, a descriptive analysis (mean [M] and standard deviation [SD]) of the students’ responses to the SWLS was performed. In addition, a Student’s t-test was used to assess whether there were significant differences between the means of women and men.

## 3 Results

### 3.1 Exploratory factor analysis using nA subsample

An analysis of the data suggests that the SWLS items showed a distribution within the limits of normality. According to Arenas et al. (2023), items are normally distributed variables when their asymmetry is less than 2 and kurtosis less than 7, and the analysis carried out in the present study showed maximum values of 0.705 for asymmetry and −1.18 for kurtosis (see Table 1).

**Table 1:** Descriptive statistics and factor loading of SWLS items.

Items	Mean	Standard deviation	Asymmetry	Kurtosis	Factor 1
Item 1	4.85	1.49	−0.644	−0.086	0.753
Item 2	4.99	1.35	−0.634	0.113	0.808
Item 3	5.12	1.48	−0.705	−0.005	0.923
Item 4	4.94	1.50	−0.580	−0.244	0.822
Item 5	4.36	1.91	−0.184	−1.18	0.645
Total variance					63.2%

The main tests of sampling adequacy were satisfactory (KMO measure = 0.863; Bartlett’s sphericity test: X^2^ [10] = 6745.794; *p* < 0.001), so it was considered pertinent to carry out the respective EFA.

The EFA of the 5 items of the Satisfaction with Life scale using the principal axis factoring method with oblimin rotation yielded a single-factor solution that accounts for 63.2% of the total variance of the test. All loadings were values greater than 0.60 ranging between 0.645 and 0.923 (see Table 1).

### 3.2 Confirmatory factor analysis using nB subsample

To determine the factor structure of the scale, the goodness-of-fit indices of two different models were compared: (1) One factor, based on the results obtained in the EFA and the consistent results in the CFA of several previous studies including the original author of the scale and his collaborators (Diener et al., 1985), and (2) two factors, satisfaction with life with present achievements (items 1, 2 and 3) and past achievements (items 4 and 5) reported by Bagherzadeh et al. (2018), Pavot and Diener (1993) and Sachs (2003).

**Figure 1:**
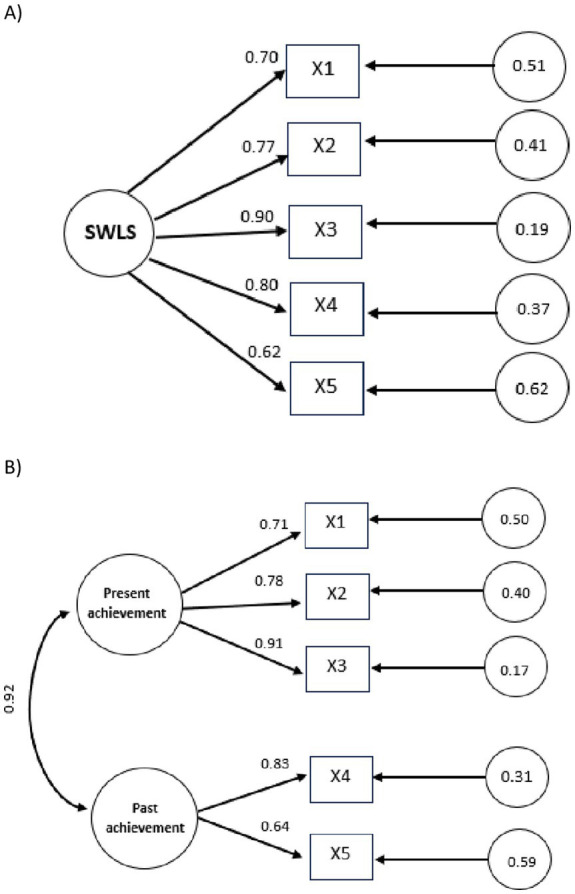
Diagram of the models resulting from the confirmatory factor analysis of the SWLS, Single-factors model **(A)**, Two-factor model **(B)**.

Based on the fit indices, both models could represent the data observed for the sample of university students. Although the two-factor or two-dimensional model (present achievements and past achievements) fits better to the empirical evidence compared to model 1 (unifactorial; see Table 2), the correlation between these two dimensions is very high *r* = 0.92, which makes this differentiation questionable. In addition, in the first dimension (present achievements), it loaded three items and in the other (past achievements) only two items (see Figure 1B). It is evident that a dimension with only two items should be avoided, if possible, when defining a construct (Sachs, 2003). Therefore, the one-factor model 1, which presented an optimal fit (*X^2^/df* = 3.79; CFI = 0.998; TLI = 0.996; SRMR = 0.023; RMSEA = 0.030) and is more parsimonious and consistent with the theoretical framework associated with the development of the scale as a unidimensional measure of general satisfaction with life, is proposed as the final solution for the data of the Ecuadorian students (See Figure 1A).

**Table 2:** Fit indices of the models used in confirmatory factor analysis.

Model	*X^2^ / df*	CFI	TLI	SRMS	RMSEA
1: One factor	3.79	0.998	0.996	0.023	0.030
2: Two factors	4.79	0.999	0.998	0.017	0.021

X^2^ / df, Chi-square by degrees of freedom; CFI, Bentler Comparative Fit Index; TLI, Tucker-Lewis coefficient; SRMR, Standardized root mean square residual value, RMSEA, Root mean square error of approximation.

Regarding convergent internal validity, the result obtained was satisfactory (AVE = 0.553), which suggests that the construct explains a significant proportion of the variance observed in the responses to the items, evidencing an adequate explanatory capacity.

### 3.3 Invariance analysis

The single-factor model was selected for the following SWLS factorial invariance analysis for the total sample and by sex. The results are shown in Table 3, where the configural invariance (M1) can be observed showing good fit indicators (CFI = 0.999 and RMSEA = 0.016). The metric invariance (M2) resulted in good fit indices (CFI = 1.000; RMSEA = 0.009), similar to the M1 values because they presented minimal differences (ΔCFI = 0.001 and ΔRMSEA = −0.0007). The factor loadings did not vary between both sexes, so the covariances can be compared. The scalar invariance (M3) showed indices equal to the previous model (CFI = 0.999; RMSEA = 0.014) with minimal differences (ΔCFI = −0.001 and ΔRMSEA = 0.005). Invariance between thresholds is accepted. The strict invariance (M4) reflected a good fit (CFI = 0.998; RMSEA = 0.018) with minimal differences (ΔCFI = −0.001 and ΔRMSEA = 0.004), so the invariance of residuals is verified. The combined results indicate factorial invariance of SWLS in both sexes.

**Table 3:** Factorial invariance of the SWLS for the total sample and by sex.

Model	*χ* ^2^	Df	C-M	Δχ^2^	Δ*df*	CFI	ΔCFI	SRMR	RMSEA	ΔRMSEA
Total	18.930	5	–	–	–	0.998	–	0.023	0.030	–
Men	5.485	5	–	–	–	1.00	–	0.022	0.010	–
Women	7.318	5	–	–	–	0.999	–	0.023	0.019	–
M1	12.802	10	–	–	–	0.999	–	0.023	0.016	–
M2	15.193	14	M2-M1	2.391	4	1.00	0.001	0.025	0.009	−0.007
M3	21.713	18	M3-M2	6.520	4	0.999	−0.001	0.025	0.014	0.005
M4	31.185	23	M4-M3	9.472	5	0.998	−0.001	0.030	0.018	0.004

χ^2^, chi-square analysis; df, degrees of freedom; C-M, comparison of factorial invariance models; CFI, Bentler Comparative Fit Index; SRMR, standardized root mean square residual; RMSEA, root mean square error of approximation; Δ, increase; Models, M1, configural model; M2, metric model; M3, scalar model; M4, strict model.

### 3.4 Internal consistency and divergent validity

The internal consistency for the SWLS total score was satisfactory (*ω* = 0.84), which guarantees the reliability of the scale. Since the model was not bifactorial, there was no need to calculate the hierarchical omega coefficient. Likewise, there was no correlation between the errors, so the omega coefficient did not have to be corrected either (Vizioli, 2021).

Regarding divergent validity, the correlation between the scores of the Satisfaction with Life Scale (SWLS) and the scores of the Patient Health Questionnaire (PHQ-9) that measures depression and the Perceived Stress Scale (PSS-10) was analyzed with the complete sample (*N* = 4,524).

The results in Table 4 indicate that satisfaction with life exhibits a strong negative correlation with depressive symptoms and perceived stress, supporting adequate divergent validity.

**Table 4:** Correlation matrix between SWLS and other mental health-related measures.

	Depression	Perceived stress
SWLS (Satisfaction with life)	−0.505**	−0.525**

**p < 0.001.

### 3.5 Descriptive analysis

Taking the scale as unifactorial, a summed score of all the items was calculated. The normality calculated through the asymmetry and kurtosis coefficients showed compliance with this assumption (−1.1; Zerpa et al., 2023). Regarding the analysis of this score, the university students evaluated presented moderately high satisfaction scores (M = 24.29; As = −0.484), with moderate dispersion around the mean and a platykurtic distribution (SD = 6.33; K = −0.164).

With homogeneity of variance between groups, evaluated by Levene’s test (*F* = 3.08; df = 1, 4,522; *p* = 0.08), it was found that there were no significant differences between men (*M* = 24.38; *SD* = 6.44) and women (*M* = 24.22; *SD* = 6.23) according to Student’s t-test [t(4,522) = 0.842, *p* = 0.400].

## 4 Discussion

The main objective of the present study was to analyze the psychometric properties and factor structure of the Spanish version of the SWLS in university students in Ecuador considering the lack of a validated instrument that measures satisfaction with life in this population. According to the findings, it was empirically verified, through construct validity (EFAyo lo and CFA), factorial invariance, AVE, reliability (omega coefficient) and divergent validity (Pearson correlations), that the SWLS is a reliable and valid tool to measure satisfaction with life in university students in the Ecuadorian context.

Regarding the validity evidence based on the SWLS structure, the EFA was carried out, which determined a unidimensional factor structure that explains 63.2% of the total variance of the SWLS, with satisfactory factor loading estimation. These results are consistent with those reported in studies developed in university students in Colombia (Vinaccia Alpi et al., 2019), the Dominican Republic (Zerpa et al., 2023) and in the general population in Argentina (Mikulic et al., 2019) that yielded a single-factor model that explains 62.3, 65, 54.35 and 64.58% of the total variance of the scale, respectively.

Subsequently, in the confirmatory factor analysis, two models were contrasted: one with one factor and another with two factors (present and past achievements), and it was found that both models could represent the data observed for the sample of Ecuadorian university students. However, the single-factor model was considered the final solution since, in addition to presenting an optimal fit, it is more parsimonious and theoretically consistent with the development of the SWLS scale. This single-factor structure has been reported in the cross-cultural study by Jang et al. (2017) carried out with adults from 26 countries on four continents and with university students in Chile (Bagherzadeh et al., 2018), Spain (Delgado-Lobete et al., 2020), the Dominican Republic (Zerpa et al., 2023) and, specifically, in Ecuador (Arias and García, 2018; Schnettler et al., 2017).

Regarding the factorial invariance of the SWLS for the total sample and by sex, the results indicate that the one-factor model remained invariant between men and women, thus demonstrating that the SWLS is a safe tool to apply to university students of both sexes. This was also found in the Ecuadorian adult population (Arias and García, 2018; Schnettler et al., 2017). Therefore, any differences in these groups that may be found through this scale are due to real differences in levels of satisfaction with life and not to an artifact of measurement error.

On the other hand, it was found that SWLS maintained satisfactory values in the omega coefficient *ω* = 0.84, which indicates that it is a reliable instrument. These results are similar to those reported by other authors, for example, the value of the omega coefficient is similar to that reported in the study by Areepattamannil and Bano (2020) in India and to that of the research by Bagherzadeh et al. (2018) in Chile, where ω = 0.80 was obtained. Although the alpha coefficient was not used to calculate reliability, previous studies that do use it report values similar to the omega obtained in the present research (Arias and García, 2018; Garcia et al., 2021; González Rivera and Rosario Rodríguez, 2020; Merino et al., 2021; Mikulic et al., 2019; Nooripour et al., 2023; Schnettler et al., 2017).

Regarding the divergent validity of the SWLS, it was found that the scores of this scale correlated in a moderately high and negative way with the constructs of depression and perceived stress. These findings are consistent with those of previous studies, in which the negative association between satisfaction with life and depressive symptoms (López-Guerra et al., 2022; Nooripour et al., 2023) and perceived stress (Nooripour et al., 2023) was observed. The evidence of such associations suggests that satisfaction with life could be a protective factor for mental health.

Finally, the levels of satisfaction with life of the students participating in the study are moderately high; in this way, the tendency of the participants in this study is toward a positive response about satisfaction with their lives, which does not coincide with findings in adolescents (Avila and Cañas-Lucendo, 2023), but does with the results obtained in the adult Ecuadorian population, whether they are university students or not (Arias and García, 2018; Hidalgo-Fuentes et al., 2021; Moreta-Herrera et al., 2018; Ubilla et al., 2020). This indicates that in general terms adult Ecuadorians are moderately happy and consider their life excellent. It remains to delve deeper into the possible differences by evolutionary cycle and those cases with lower levels of satisfaction.

On the other hand, no differences were found in satisfaction with life between men and women. This finding is consistent with the studies of Pavot and Diener (1993), Vázquez et al. (2013), Vinaccia Alpi et al. (2019) and Zerpa et al. (2023), in latitudes other than Ecuador, and in Ecuador in university students (Hidalgo-Fuentes et al., 2021). Research in the Ecuadorian adult non-university population and elderly population does find differences by sex. However, the results are contradictory (Arias and García, 2018; Moreta-Herrera et al., 2018), so these differences should continue to be evaluated, while also considering age.

### 4.1 Limitations and practical implications

Is important to point out some limitations of the study. Firstly, the data must be interpreted with caution since the sample was non-probabilistic and only comprised university students, which makes it difficult to generalize the findings to the rest of the youth population. Furthermore, factorial invariance due to sociocultural factors, which are important in Ecuador, was not considered. Therefore, it is suggested that studies be carried out to further explore the psychometric properties of the scale in other Ecuadorian samples from different socioeconomic strata, ethnicities, and age groups. Secondly, the design of this study is cross-sectional, so the measurement of test–retest reliability could be assessed in future research through a longitudinal design. Thirdly, only discriminant validity was evaluated, and it is important to assess other types of validity (convergent, criterion, predictive, for example). Finally, the use of tools such as the SWLS may be subject to certain inaccuracies in data collection due to recall bias and social desirability. For future research, the use of scales intended to measure and control potential biasing sources that may influence the results is suggested (Rosenmanm et al., 2011).

Despite the limitations mentioned, this study lays the foundation for future research on satisfaction with life using SWLS in the Ecuadorian population. Satisfaction with life in university students is closely related to important aspects of the developmental stage of these young people, such as achievement of goals and definition of objectives, as well as cognitive, emotional, and motivational aspects that influence subjective well-being, such as self-acceptance, autonomy, self-determination, and an optimistic view of life (Chan Chi, 2021; García-Alandete et al., 2018). Likewise, it affects the ability to cope with adversity, responsibility, and academic performance, which are elements directly linked to the university experience, so having an instrument to measure life satisfaction is very important.

The findings confirm that the SWLS presents adequate indicators of reliability and validity in young university students in Ecuador, which positions it as a useful and appropriate tool for the evaluation of satisfaction with life in this context.

Furthermore, the results obtained support the use of the SWLS as a brief, easy-to-administer, and freely available instrument for measuring satisfaction with life in university students. Its implementation in research and practice within the university setting can significantly contribute to the assessment of this construct, allowing the design of interventions aimed at improving and promoting satisfaction with life and, ultimately, the well-being of students in the academic context. This in turn would allow the development of strategies that strengthen their psychological and academic adjustment.

## 5 Conclusion

The findings of this research indicate that the Satisfaction with Life scale reflects adequate indicators of validity and reliability that were consistent with previous studies, which is why it is considered an excellent instrument that can be used to measure satisfaction with life in Ecuadorian university students, as well as for the development of future research in the field of positive psychology in Ecuador.

## Data Availability

The raw data supporting the conclusions of this article will be made available by the authors, without undue reservation.
